# Synthesis and characterization of bis(4-amino-2-bromo-6-methoxy)azobenzene derivatives

**DOI:** 10.3762/bjoc.15.296

**Published:** 2019-12-30

**Authors:** David Martínez-López, Amirhossein Babalhavaeji, Diego Sampedro, G Andrew Woolley

**Affiliations:** 1 Departamento de Química, Universidad de La Rioja, Centro de Investigación en Síntesis Química (CISQ), Madre de Dios, 53, 26006 Logroño, Spain; 2 Department of Chemistry, University of Toronto, 80 St. George St., Toronto, M5S 3H6, Canada

**Keywords:** azobenzene, azonium, molecular switches, *ortho* substitution, photoisomerization, photoswitch, visible light

## Abstract

Aminoazobenzene derivatives with four *ortho* substituents with respect to the N–N double bond are a relatively unexplored class of azo compounds that show promise for use as photoswitches in biology. Tetra-*ortho*-methoxy-substituted aminoazobenzene compounds in particular can form azonium ions under physiological conditions and exhibit red-light photoswitching. Here, we report the synthesis and characterization of two bis(4-amino-2-bromo-6-methoxy)azobenzene derivatives. These compounds form red-light-absorbing azonium ions, but only under very acidic conditions (pH < 1). While the low p*K*_a_ makes the azonium form unsuitable, the neutral versions of these compounds undergo *trans*-to-*cis* photoisomerization with blue-green light and exhibit slow (τ_1/2_ ≈ 10 min) thermal reversion and so may find applications under physiological conditions.

## Introduction

**Figure 1:**
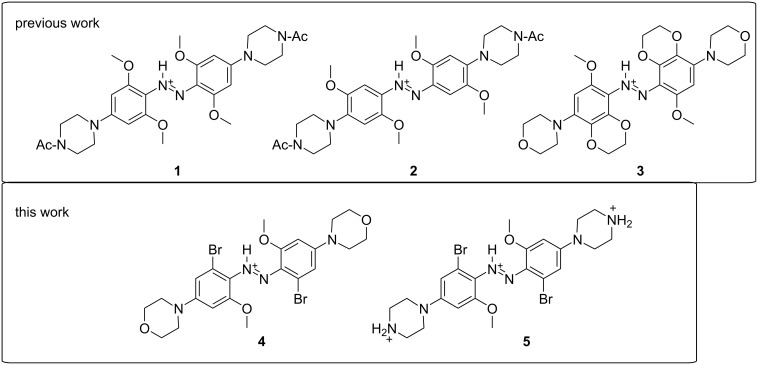
Structures of azonium ions studied.

The application of photoswitches to control biological targets has been a driving force for the development of photoswitches that operate at wavelengths that are compatible with cells and tissues. While many classes of photoswitches are known [1–2], few of these are easily adaptable to controlling targets, such as proteins [3], while simultaneously exhibiting robust photochemistry in the red or near-infrared (NIR) regions of the spectrum [4–7]. Azonium ions – protonated forms of azobenzenes – have recently been found to exhibit photoswitching properties suitable for in vivo use [8–10]. Typically, the formation of azonium ions from aminoazobenzenes occurs at pH < 3.5 [11–12], however, the p*K*_a_ of the *trans*-azonium ion **1** is ca. 7.5 in aqueous solution (Figure 1) [9–10]. The elevated p*K*_a_ of **1** has been attributed to resonance stabilization of the azonium cation together with intramolecular H-bonding between the azonium proton and methoxy groups in *ortho*-position to the azo double bond [10]. Since the azonium ion **1** forms under physiological conditions, i.e., at neutral pH value in an aqueous solution, it is useful as a photoswitch for the photocontrol of biomolecules [6]. It absorbs red light, undergoes *trans*-to-*cis* photoisomerization, and relaxes to the *trans* isomer in the dark on the timescale of seconds so that pulses of red light can be used to drive multiple isomerization cycles [10]. Efforts to apply **1** to photocontrol protein–protein interactions have recently been reported [13].

Despite the usefulness of **1** as a photoswitch, compounds undergoing photoisomerization at longer wavelengths (>700 nm) would be valuable since the penetration of light through tissue is enhanced in the NIR window [7]. Longer-wavelength absorption is achieved by compounds **2**, **3**, and related derivatives (Figure 1) [9]. However, the lifetime of thermal reversion of the *cis* isomer of **2** is only ≈1 ms at a neutral pH value, and the p*K*_a_ for *trans*-azonium ion formation is ca. 2.6. The low p*K*_a_ was attributed to a steric clash between the methoxy groups in *meta*-position to the azo double bond and the six-membered morpholino ring [9]. The rapid thermal reversion was attributed to the removal of two *ortho*-methoxy groups, leading to diminished steric strain in the transition state for reversion [9]. In compound **3**, all four *ortho*-positions are substituted, and the *meta*-oxygen substituents are part of dioxane rings so that the steric clash with the *para*-amino substituents is reduced. Compound **3** and derivatives with pyrrolidino groups in the *para*-positions were shown to be effective NIR switches, undergoing isomerization with 720 nm light under physiological conditions [8]. While the photoswitching properties of **3** are suitable for the use in biological systems, the overall size of **3** may limit the possibilities for the use as a component of photopharmaceutical agents. Currently, most photopharmaceutical agents are constructed by adding a photoswitchable unit to a pharmacophore [14–15], thereby significantly increasing the size of the compound and decreasing its potential as a drug. Therefore, we were interested in exploring other substitution patterns for these aminoazobenzene derivatives. To allow for the possibility of intramolecular H-bond formation, we wished to retain at least one methoxy group. To reduce the rate of thermal reversion, only derivatives with substituents in all four *ortho*-positions were considered. Time-dependent density functional theory (TD-DFT) calculations were used to predict the absorption wavelengths of possible derivatives. Based on these considerations, we carried out the synthesis and photochemical characterization of compounds **4** and **5**.

## Results and Discussion

### Computational chemistry

**Figure 2:**
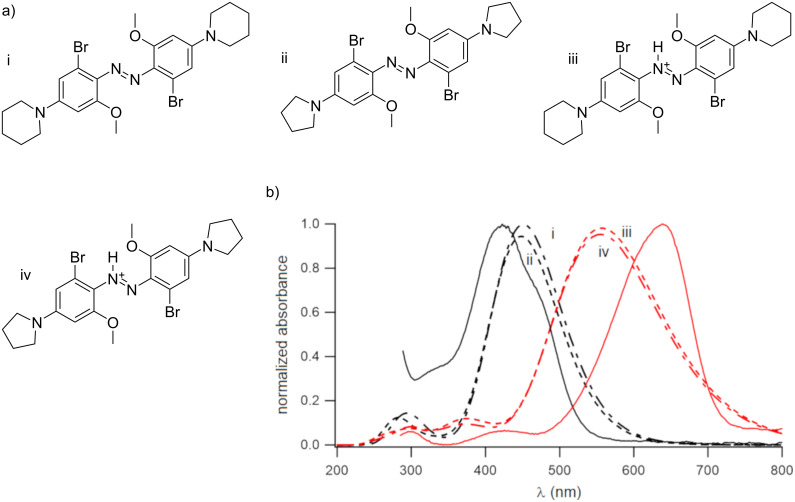
a) Structures of model compounds used for computations (see Experimental section; in calculations, water was set as the solvent). b) Calculated spectra of neutral forms i and ii (black spectra) and azonium forms iii and iv (red spectra), carrying either *para*-piperidino (i and iii, dashed lines) or *para*-pyrrolidino (ii and iv, dash-dotted lines) substituents. Solid lines show experimental spectra of **4** in DCM (*c* ≈ 15 µM) without (black) or with TFA added (red).

Calculations were performed using density functional theory (DFT) methods (B3LYP/6-31+G**) to optimize geometry, and TD-DFT with a Solvation Model based on Density (SMD) to calculate absorption wavelength maxima. These computational methods have been used successfully with related compounds [16–17]. The relative stability of different conformations of the molecule was calculated, i.e., with the methoxy substituents on the same or on the opposite side of the N–N double bond. The conformation where both methoxy groups were on the opposite side was found to be the most stable one, although the conformation where both methoxy groups were on the same side was also predicted to be significantly populated at 20 °C (see Supporting Information File 1). Calculating the effect of the substitution pattern on the p*K*_a_ value is problematic [18] and was not attempted here. Figure 2 shows calculated structures and spectra of the neutral forms of simplified models of **4** and **5**, with either a pyrollidino or piperidino substituent in *para*-position, as well as the corresponding azonium ions. Calculations indicated that the nature of the respective amino substituent did not have a large effect on the positions of wavelength maxima. These models predicted that compounds **4** and **5** should absorb at longer wavelengths than **1** [10]. Figure 2 also shows experimental spectra, which are discussed below.

### Synthesis

**Scheme 1:**
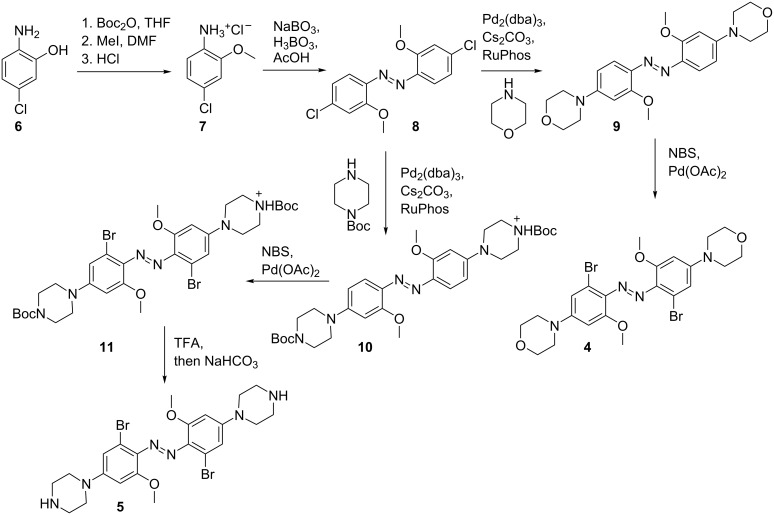
Synthesis of bis(4-amino-2-bromo-6-methoxy)azobenzene compounds.

The overall synthetic route that was taken is shown in Scheme 1. The azo compound **8**, carrying two *ortho-*methoxy groups, was prepared from **7** using an oxidative coupling approach [19]. The *para*-chloro substituents were then replaced by amino substituents using a Buchwald–Hartwig coupling [20]. Since calculations predicted that 5- and 6-membered rings would have similar effects on the positions of the absorption maxima, we opted to use 6-membered rings, specifically a morpholino substituent and a piperazino substituent in an attempt to enhance water solubility. Late-stage functionalization of the *ortho*-position was carried out through a palladium(II)-catalyzed C–H activation, resulting in *ortho*-brominated azobenzenes [21].

### Photochemical characterization

Despite the morpholino substituent, compound **4** was found to be insoluble in water. We therefore dissolved **4** in DCM to obtain the UV–vis spectrum of the neutral form. Addition of TFA to this solution produced the corresponding azonium ion, and the spectra of the neutral and azonium forms of **4** are shown as solid lines in Figure 2. Observed absorption maxima were at 426 (neutral form) and 640 nm (azonium form). While the observed absorption maximum wavelength was close to that predicted for the neutral form, the observed absorption maximum wavelength of the azonium ion was significantly higher than predicted, although the signal’s tail was less pronounced. The absorption maximum wavelength of the azonium ion was also higher than that observed for compound **1** [10], as predicted.

**Figure 3:**
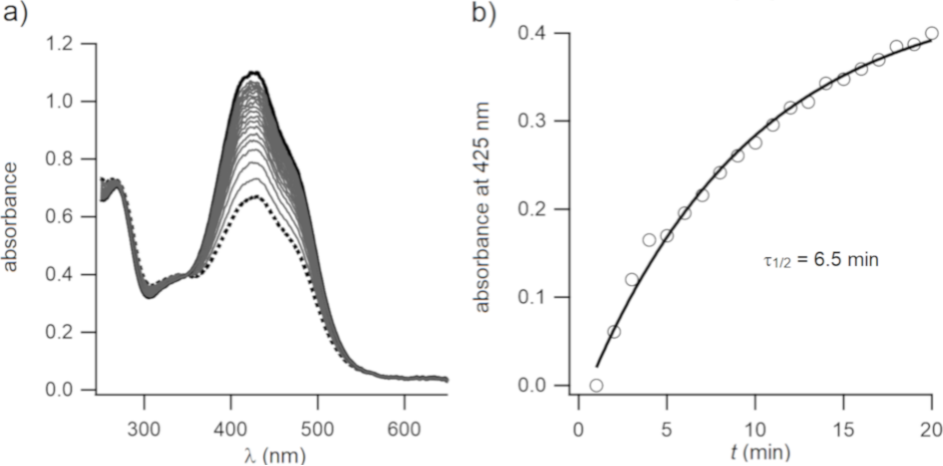
a) UV–vis spectra of **4** in DCM (ca. 15 µM) at the PSS and 440 nm irradiation (thick dotted line; ca. 60% *Z*-isomer), and during thermal reversion to the dark-adapted state (solid black line). b) Time course of thermal reversion at 22 °C. Data were fitted to a single exponential decay equation (solid line).

We confirmed that the neutral form of **4** underwent photoisomerization. Exposure of a solution of **4** in DCM to 440 nm light led to a photostationary state (PSS) in which the absorbance at 440 nm was diminished and that at 330 nm slightly enhanced (Figure 3). An estimated PSS of ca. 60% *Z*-isomer was calculated as described in the Experimental section. Thermal relaxation from the PSS was monitored by UV–vis spectroscopy, recording a spectrum every minute. As shown in Figure 3, a half-life of 6.5 minutes was obtained at room temperature.

To enhance the compounds’ water solubility, the morpholino substituents were replaced by piperazino groups because the secondary amino groups on the piperazino units were expected to have p*K*_a_ values near 10 [22], and so should be protonated at neutral pH, creating a doubly charged species. As anticipated, compound **5** was found to be much more water-soluble than **4**.

**Figure 4:**
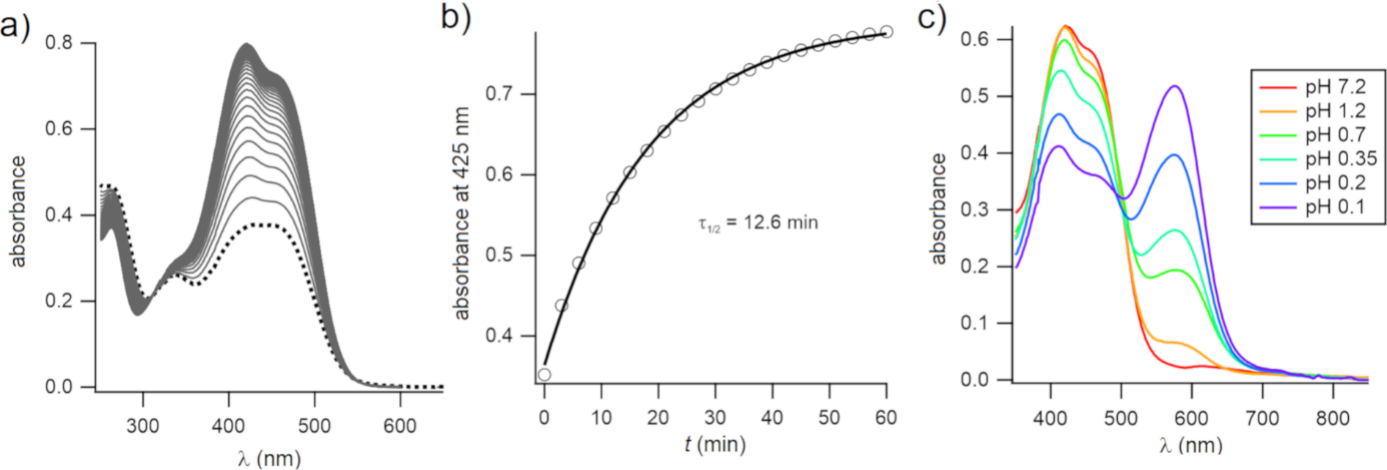
a) UV–vis spectra of **5** in aqueous solution (*c* ≈ 15 µM, 5% methanol, pH 7) at the PSS and 440 nm irradiation (thick dotted line; ca. 60% *Z*-isomer) and during thermal reversion to the dark-adapted state (solid black line). b) Time course of thermal reversion at 22 °C. Data were fitted to a single exponential decay equation (solid line). c) Spectra of an aqueous solution of **5** upon addition of hydrochloric acid, as indicated by pH values. The wavelength of the absorption maximum of the azonium ion was 575 nm.

The UV–vis spectrum of **5** at a neutral pH value is shown in Figure 4. Irradiation with blue light at 440 nm produced the PSS (dotted line). Thermal reversion from the *cis* isomer was monitored by UV–vis spectroscopy, recording a UV–vis spectrum every 3 minutes, and a half-life of 12.6 minutes was obtained.

The formation of the azonium ion of **5** in aqueous solution was explored thereafter. Addition of hydrochloric acid to a neutral solution of **5** was carried out. Spectra at different pH values are shown in Figure 4c. As can be seen, the formation of the azonium ion of compound **5** required strongly acidic conditions. Even at pH ≈ 0.1, a substantial fraction of the neutral species was still present, implying that the p*K*_a_ value of the azonium ion was below ca. 0.2. This p*K*_a_ value was at least 7 pH units lower than for the tetra-*ortho*-methoxy compound **1**. As noted above, the presence of the piperazino amino groups made compound **5** doubly positively charged at a neutral pH value. This feature was expected to reduce the p*K*_a_ value of the azonium ion through electrostatic effects [23]. The first p*K*_a_ value of substituted piperazines falls in the range ca. 4–6 (vs 9–10 for the second p*K*_a_ value) [22]. In addition, the electron-withdrawing bromine atoms were also expected to lower the azonium ion’s p*K*_a_ value (the p*K*_a_ values of 2-bromobenzoic acid and unsubstituted benzoic acid are 2.85 and 4.2, respectively [24]). While electrostatic effects on the p*K*_a_ value could be ameliorated (i.e., by adding negatively charged groups), this would place additional constraints on the general applicability of the compounds. In addition, unlike compound **4**, the wavelength of the absorption maximum of the azonium ion of **5** was not red-shifted relative to that of **1**. Conceivably, the wavelength of maximum absorbance was affected by the charged piperazino groups; calculations were done with uncharged piperidino substituents, as shown in Figure 2. Nevertheless, the lack of a red-shift, combined with the significantly lowered p*K*_a_ value, made **5** unsuitable as an azonium photoswitch under physiological conditions.

Despite this undesired effect on the p*K*_a_ value, the steric bulk introduced by the bromine substituents did appear to slow thermal relaxation of the neutral (unprotonated) azo forms of these compounds. Species **5** could be switched with blue and green light under physiological conditions and be thermally relaxed with a half-life of 12 minutes. This relaxation rate was substantially lower than other blue-green-absorbing azo compounds without substituents in all four *ortho*-positions relative to the azo unit, which showed half-lives ranging from 50 ms [25] to a few seconds [26–27]. Instead, compounds **4** and **5** exhibited photoswitching properties similar to those reported for tetra-*ortho*-thiol-substituted azobenzenes [17].

## Conclusion

Substitution of *p*-aminoazobenzene with *ortho*-bromo and *ortho*-methoxy groups (i.e., a 2-bromo-6-methoxy substitution pattern) was found to lower the p*K*_a_ of the azonium ion such that it fell outside the normal physiological range. The neutral version of this compound nevertheless underwent *trans*-to-*cis* photoisomerization in the presence of blue-green light and exhibited slow thermal relaxation (τ_1/2_ ca. 10 min).

## Experimental

### General

All commercial materials (solvents, reagents, and substrates) were used as received. SilicaFlash silica gel, P60, 40–63 µm particle size (SiliCycle) was used for column chromatography. High-performance liquid chromatography was performed on a PerkinElmer Series 200 pump with a Waters 2487 Dual λ Absorbance Detector connected to an eDAQ PowerChrom 280 recorder. One-dimensional ^1^H and ^13^C NMR spectra were recorded on a Varian UnityPlus 500 MHz or Varian Mercury 400 MHz spectrometer. Chemical shifts are reported in ppm, and the signals were referenced to residual undeuterated solvent signals. Mass spectra were recorded using an Agilent 6538 mass spectrometer with a Q-TOF ionization source or a JEOL AccuTOF mass spectrometer with a DART ionization source.

### Synthesis and characterization

**4-Chloro-2-methoxyanilinium chloride (7):** (1) 2-Amino-5-chlorophenol (**6**, 3.5 mmol, 500 mg) was dissolved in THF at room temperature. Then, Boc_2_O (7 mmol, 1.52 g) was added and the resulting mixture was stirred for 18 h at room temperature. The reaction progress was monitored by TLC. When the reaction was complete, the solvent was removed under reduced pressure, resulting in a yellowish oil. This was purified by column chromatography using hexane/ethyl acetate, 4:1, v/v as eluent. The product, phenol *tert*-butyl (4-chloro-2-hydroxyphenyl)carbamate, was used directly in the next step. (2) *tert*-Butyl (4-chloro-2-hydroxyphenyl)carbamate (2 mmol, 500 mg) was dissolved in DMF. Then, an aqueous solution of K_2_CO_3_ (6 mmol, 828 mg) was added, together with methyl iodide (Caution: toxic, potential carcinogen; 6 mmol, 851 mg). The mixture was stirred for 18 h until the starting reagent was consumed. Then, the solvent was removed under reduced pressure, resulting in a brown oil. This was poured into water and extracted three times with DCM. The organic fractions were collected and evaporated under reduced pressure. The product, *tert*-butyl (4-chloro-2-methoxyphenyl)carbamate, was used directly in the next step. (3) *tert*-Butyl (4-chloro-2-methoxyphenyl)carbamate (400 mg, 1.16 mmol) was dissolved in 3 mL of ethyl acetate. Then, 9 mL of fuming hydrochloric acid were added dropwise to the reaction mixture with vigorous stirring. The resulting mixture was stirred for 1 hour. Then, the solvent was evaporated under reduced pressure, giving a brown oil. This was added to 30 mL of hexane in an ice bath to precipitate the title compound. Finally, the product **7** (420 mg, 77% over three steps) was isolated by filtration. ^1^H NMR (400 MHz, methanol-*d*_4_) δ 7.36 (d, *J* = 8.4 Hz, 1H), 7.30 (d, *J* = 2.1 Hz, 1H), 7.11 (dd, *J* = 8.4, 2.1 Hz, 1H), 3.99 (s, 3H).

**(*E*)-1,2-Bis(4-chloro-2-methoxyphenyl)diazene (8):** 4-Chloro-2-methoxyanilinium chloride (**7**, 2.5 mmol, 400 mg) was dissolved in acetic acid (2 mL), and boric acid (2.11 mmol, 135 mg) was added to the mixture, followed by sodium perborate (2.5 mmol, 680 mg) in three portions over 15 minutes. Then, the reaction mixture was heated at 70 ºC for 18 hours. The reaction progress was monitored by TLC, and when the reaction was complete, the solvent was removed under reduced pressure. The resulting oil was poured into water and extracted with DCM three times. The organic fractions were collected, and the solvent was removed. The resulting oil was purified by column chromatography using hexane/ethyl acetate, 4:1, v/v as eluent. This way, 250 mg (32%) of **8** could be obtained. ^1^H NMR (300 MHz, chloroform-*d*) δ 7.59 (d, *J* = 8.6 Hz, 2H), 7.07 (d, *J* = 2.1 Hz, 2H), 6.98 (dd, *J* = 8.6, 2.1 Hz, 2H), 4.01 (s, 6H); ^13^C NMR (75 MHz, chloroform-*d*) δ 157.4, 141.4, 138.3, 121.2, 118.5, 113.3, 56.7; HRMS (*m*/*z*): [M + H]^+^ calcd for C_14_H_15_N_2_O_2_Cl_2_, 311.0349; found, 311.0341.

**(*E*)-1,2-Bis(2-methoxy-4-morpholinophenyl)diazene (9):** (*E*)-1,2-Bis(4-chloro-2-methoxyphenyl)diazene (**8**, 100 mg, 0.32 mmol) was dissolved in toluene in an Ace pressure tube (10.2 cm × 8 mm). Then, morpholine (84 mg, 0.96 mmol), tris(dibenzylideneacetone)dipalladium(0) (29.3 mg, 0.032 mmol), RuPhos (29.8 mg, 0.064 mmol), and cesium carbonate (302.4 mg, 0.96 mmol) were added. The mixture was heated at 100 ºC in the pressure tube for 24 hours. The reaction progress was monitored by TLC until the starting reagent was consumed. The solvent was evaporated under reduced pressure, and the resulting oil was extracted with DCM three times. The resulting crude product was purified by column chromatography using hexane/ethyl acetate, 1:4, v/v as eluent to obtain 95 mg (72%) of **9**. ^1^H NMR (400 MHz, chloroform-*d*) δ 7.71 (s, 2H), 6.48 (m, 4H), 4.01 (s, 6H), 3.87 (m, 8H), 3.28 (m, 8H); ^13^C NMR (101 MHz, chloroform-*d*) δ 158.1, 154.0, 136.7, 118.5, 107.7, 98.9, 66.9, 56.7, 48.6; HRMS (*m*/*z*): [M + H]^+^ calcd for C_22_H_29_N_4_O_4_, 413.2189; found, 413.2186.

**(*E*)-1,2-Bis(2-bromo-6-methoxy-4-morpholinophenyl)diazene (4):** (*E*)-1,2-Bis(2-methoxy-4-morpholinophenyl)diazene (**9**, 30 mg, 0.07 mmol) was dissolved in DCM. To this solution, palladium acetate (1.6 mg, 0.007 mmol) was added, and the resulting mixture was stirred for 15 minutes. Then, *N*-bromosuccinimide (28.6 mg, 0.16 mmol) was added to the reaction. The reaction mixture was stirred for additional 30 minutes until completed. The solvent was evaporated under reduced pressure, and the resulting oil was purified by column chromatography using hexane/ethyl acetate, 1:2, v/v as eluent to obtain 29 mg (70%) of **4**. ^1^H NMR (300 MHz, chloroform-*d*) δ 7.91 (s, 2H), 6.67 (s, 2H), 4.03 (s, 6H), 3.89 (m, 8H), 3.17 (s, 8H); ^13^C NMR (101 MHz, chloroform-*d*) δ 157.3, 153.7, 138.8, 122.6, 110.8, 104.9, 67.0, 58.6, 51.9; HRMS (*m*/*z*): [M + H]^+^ calcd for C_22_H_27_Br_2_N_4_O_4_, 569.0393; found, 569.0393.

**Di-*tert*-butyl 4,4'-(diazene-1,2-diyl)bis(3-methoxy-4,1-phenylene)-(*E*)-bis(1λ^4^-piperazine-1-carboxylate) (10):** (*E*)-1,2-Bis(4-chloro-2-methoxyphenyl)diazene (**8**, 100 mg, 0.32 mmol) was dissolved in toluene in a pressure tube. Then, 1-Boc-piperazine (180 mg, 0.96 mmol), tris(dibenzylideneacetone)dipalladium(0) (29.3 mg, 0.032 mmol), RuPhos (29.8 mg, 0.064 mmol), and cesium carbonate (302.4 mg, 0.96 mmol) were added. The mixture was heated at 100 ºC in a pressure tube for 36 hours. The reaction progress was monitored by TLC until the starting reagent was consumed. The solvent was evaporated under reduced pressure, and the resulting oil was extracted with DCM three times. The final mixture was purified by column chromatography using hexane/ethyl acetate, 1:3, v/v as eluent to obtain 130 mg (65%) of the product **10**. ^1^H NMR (400 MHz, DCM-*d*_2_) δ 7.57 (m, 2H), 6.50 (m, 4H), 3.99 (s, 6H), 3.58 (m, 8H), 3.29 (m, 8H), 1.47 (s, 18H).

**Di-*tert*-butyl 4,4'-(diazene-1,2-diyl)bis(3-bromo-5-methoxy-4,1-phenylene)-(*E*)-bis(1λ^4^-piperazine-1-carboxylate) (11):** Di-*tert*-butyl 4,4'-(diazene-1,2-diyl)bis(3-methoxy-4,1-phenylene)-(*E*)-bis(1λ^4^-piperazine-1-carboxylate) (**10**, 30 mg, 0.04 mmol) was dissolved in DCM, palladium acetate (1 mg, 0.004 mmol) was added, and the resulting mixture was stirred for 15 minutes. Then, *N*-bromosuccinimide (22 mg, 0.1 mmol) was added to the reaction. The reaction mixture was stirred for additional 30 minutes until completion. The solvent was evaporated under reduced pressure and the resulting oil was purified by column chromatography using hexane/ethyl acetate, 1:1, v/v as eluent to obtain 19 mg (50%) of the product **11**. ^1^H NMR (400 MHz, DCM-*d*_2_) δ 7.74 (s, 2H), 6.61 (s, 2H), 3.93 (s, 6H), 3.53 (m, 8H), 3.00 (m, 8H), 1.39 (s, 18H); ^13^C NMR (126 MHz, DCM-*d*_2_) δ 152.9, 150.2, 149.4, 134.1, 117.5, 106.2, 75.1, 52.1, 47.0, 23.7; HRMS (*m*/*z*): [M + H]^+^ calcd for C_32_H_45_Br_2_N_6_O_6_, 767.1767; found, 767.1762.

**(*E*)-1,2-Bis(2-bromo-6-methoxy-4-(piperazin-1-yl)phenyl)diazene (5):** Di-*tert*-butyl 4,4'-(diazene-1,2-diyl)bis(3-bromo-5-methoxy-4,1-phenylene)-(*E*)-bis(1λ^4^-piperazine-1-carboxylate) (**11**, 15 mg, 0.02 mmol) was dissolved in 5 mL of DCM, and 0.25 mL of TFA was added. The resulting mixture was stirred for 16 hours. 1 mL of a 10% aqueous solution of sodium bicarbonate was added to neutralize the compound. Then, the solvent was evaporated to produce the product **5** (90%) without further purification needed. ^1^H NMR (400 MHz, DCM-*d*_2_) δ 7.92 (s, 2H), 6.97 (s, 2H), 4.11 (s, 6H), 3.45 (m, 8H), 3.00 (m, 8H); ^13^C NMR (126 MHz, DCM-*d*_2_) δ 158.9, 154.0, 140.2, 123.0, 111.5, 107.2, 66.9, 57.3, 45.1; HRMS (*m*/*z*): [M + H]^+^ calcd for C_22_H_29_Br_2_N_6_O_2_, 567.0719; found, 567.0713.

### UV–vis spectroscopy

UV–vis spectra were recorded on a PerkinElmer LAMBDA 35, Shimadzu UV-2401PC, or an Ocean Optics USB4000 diode array spectrophotometer. The temperature was maintained at 22 °C for all measurements (Quantum Northwest temperature controller), and 10 mm or 1.5 mm quartz cuvettes (Hellma Analytics) were used. Samples were prepared in sodium phosphate buffer, pH 7.0, or in DCM, as described, at nominal concentrations of 15 µM. The pH value was adjusted by adding microliter volumes of aq HCl or NaOH (as to not change the total volume of the sample solutions significantly). The pH values were measured directly in the samples using a combination pH electrode.

### Photoisomerization

UV irradiation was performed by placing a 365 nm LED (897-LZ440U610; LED Engin) operating at 68 mW/cm^2^ above the sample tube for 1 minute. For blue light irradiation, a 440 nm LED (Luxeon III Star LED Royal Blue Lambertian; Luxeon Star LEDs) operating at 40 mW/cm^2^ at 700 mA was used in the experiments.

### Estimates of the percentage of *cis*/*trans* isomers in PSSs

Based on NMR spectra, we assumed that the fraction of *Z*-isomer present at equilibrium in the dark was negligible. Since UV–vis spectra obtained during thermal reversion exhibited an isosbestic point, we assumed that only *E*- and *Z*-isomers contributed to the spectrum at any time. An estimate for the spectrum of pure *Z*-isomer could therefore be obtained simply by subtracting the contribution of the *E*-isomer from the spectrum of the PSS, with the restriction that the absorbance of the *Z*-isomer could not be less than zero at any wavelength. With this approach, estimated PSS values of 60% (±10%) *Z*-isomer were obtained.

### Thermal relaxation rates

The cuvette was irradiated with blue light for 1 minute, gently mixed by pipetting, then immediately capped with Parafilm to prevent evaporation, and placed inside the spectrophotometer. The sample was periodically scanned (250 to 600 nm for each scan; integration time: 2s; scan speed: 480 nm/min) in 2-minute intervals. Absorbance data vs time were then fitted to a single exponential equation to obtain thermal relaxation half-lives using Equation 1.

[1]
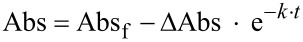


### Computational methods

DFT calculations were performed using the Gaussian 09 suite of programs at the B3LYP level of theory using a 6-31+G(d,p) basis set [28]. Optimizations were followed by harmonic oscillator frequency calculations at the same level of theory to verify the absence of imaginary frequencies. Though an exhaustive conformational search was not performed for any of the species, the following calculations were performed: To ensure that the conformation shown in Figure 2 was the thermodynamically most stable arrangement around the azo moiety, free energies for two alternative arrangements – conformer 2: with both bromine atoms on the same side of the N–N double bond and conformer 3: with a bromine atom in the position of the methoxy group that H-bonded with the azonium proton (see Supporting Information File 1) – were calculated for each compound at 298.15 K. Both alternate conformers were predicted to have higher energies in vacuo (conformer 2: 1.1 kJ/mol for the 6-membered ring and 2.2 kJ/mol for the 5-membered ring; conformer 3: 20 kJ/mol for the 6-membered ring and 19 kJ/mol for the 5-membered ring). Thus, conformer 2 was slightly higher in energy than conformer 1 and should be populated at room temperature. However, we did not carry out TD-DFT calculations for conformer 2. Second, for the neutral species, the N=N–C–C dihedral angles in the optimized structures were manually set to 20° to generate new input files. Reoptimization yielded the same structures and free energies. The optimized geometries of all structures were subjected to TD-SCF calculations using the same functionals and basis set, assuming the first 15 singlet excitations, and by applying a SMD (assuming water as the solvent) to implicitly approximate the effect of the solvent [29]. TD-SCF data were used to generate the simulated UV–vis spectra by applying a Gaussian function with 0.333 eV peak half-width at half-height to each transition. For the lowest-energy conformer of both protonated species, single point calculations were performed at B3LYP/6-31+G(d,p) with a SMD (assuming water as the solvent), and molecular orbitals corresponding to HOMO and LUMO were visualized using GaussView, with the isovalue set to 0.02. HOMO-to-LUMO transitions correspond to the longest wavelength with high oscillator strength. In general, HOMOs were found to be more delocalized than LUMOs (see Supporting Information File 1).

## Supporting Information

File 1 NMR spectra and further computational data.

## References

[1] García-Iriepa C, Marazzi M, Frutos L M, Sampedro D (2013). RSC Adv.

[2] Szymański W, Beierle J M, Kistemaker H A V, Velema W A, Feringa B L (2013). Chem Rev.

[3] Kumita J R, Flint D G, Smart O S, Woolley G A (2002). Protein Eng, Des Sel.

[4] Bléger D, Hecht S (2015). Angew Chem, Int Ed.

[5] Samanta S, Qin C, Lough A J, Woolley G A (2012). Angew Chem, Int Ed.

[6] Dong M, Babalhavaeji A, Samanta S, Beharry A A, Woolley G A (2015). Acc Chem Res.

[7] Lentes P, Stadler E, Röhricht F, Brahms A, Gröbner J, Sönnichsen F D, Gescheidt G, Herges R (2019). J Am Chem Soc.

[8] Dong M, Babalhavaeji A, Collins C V, Jarrah K, Sadovski O, Dai Q, Woolley G A (2017). J Am Chem Soc.

[9] Dong M, Babalhavaeji A, Hansen M J, Kálmán L, Woolley G A (2015). Chem Commun.

[10] Samanta S, Babalhavaeji A, Dong M-x, Woolley G A (2013). Angew Chem, Int Ed.

[11] Stoyanov S, Antonov L, Stoyanova T, Petrova V (1996). Dyes Pigm.

[12] Stoyanova T, Stoyanov S, Antonov L, Petrova V (1996). Dyes Pigm.

[13] Yasuike N, Blacklock K M, Lu H, Jaikaran A S I, McDonald S, Uppalapati M, Khare S D, Woolley G A (2019). ChemPhotoChem.

[14] Hüll K, Morstein J, Trauner D (2018). Chem Rev.

[15] Velema W A, Hansen M J, Lerch M M, Driessen A J M, Szymanski W, Feringa B L (2015). Bioconjugate Chem.

[16] Bléger D, Schwarz J, Brouwer A M, Hecht S (2012). J Am Chem Soc.

[17] Samanta S, McCormick T M, Schmidt S K, Seferos D S, Woolley G A (2013). Chem Commun.

[18] Alongi K S, Shields G C (2010). Annu Rep Comput Chem.

[19] Merino E (2011). Chem Soc Rev.

[20] Maiti D, Fors B P, Henderson J L, Nakamura Y, Buchwald S L (2011). Chem Sci.

[21] Konrad D B, Frank J A, Trauner D (2016). Chem – Eur J.

[22] Khalili F, Henni A, East A L L (2009). J Chem Eng Data.

[23] Borisenko V, Sansom M S P, Woolley G A (2000). Biophys J.

[24] Woolley E M, Tomkins J, Hepler L G (1972). J Solution Chem.

[25] Chi L, Sadovski O, Woolley G A (2006). Bioconjugate Chem.

[26] Beharry A A, Sadovski O, Woolley G A (2008). Org Biomol Chem.

[27] Sadovski O, Beharry A A, Zhang F, Woolley G A (2009). Angew Chem, Int Ed.

[28] (2009). Gaussion 09.

[29] Marenich A V, Cramer C J, Truhlar D G (2009). J Phys Chem B.

