# Supplementary material for: Synthesis and characterization of bis(4-amino-2-bromo-6-methoxy)azobenzene derivatives
Source: Beilstein J Org Chem. 2019 Dec 30;15:3000–8. doi: 10.3762/bjoc.15.296 (PMC6964645; doi:10.3762/bjoc.15.296)
Supplement: File 1 — NMR spectra and further computational data. [file Beilstein_J_Org_Chem-15-3000-s001.pdf]

BEILSTEIN JOURNAL OF ORGANIC CHEMISTRY

Supporting Information

for

Synthesis and characterization of bis(4-amino-2-bromo-6-methoxy)azobenzene derivatives

David Martínez-López, Amirhossein Babalhavaeji, Diego Sampedro and G. Andrew Woolley

Beilstein J. Org. Chem. **2019,** *15,* 3000–3008. doi:10.3762/bjoc.15.296

NMR spectra and further computational data

License and Terms: This is a supporting information file under the terms of the Creative Commons Attribution License (https://creativecommons.org/licenses/by/4.0). Please note that the reuse, redistribution and reproduction in particular requires that the authors and source are credited.
The license is subject to the *Beilstein Journal of Organic Chemistry* terms and conditions: (https://www.beilstein-journals.org/bjoc)

Synthesis/Characterization Data:

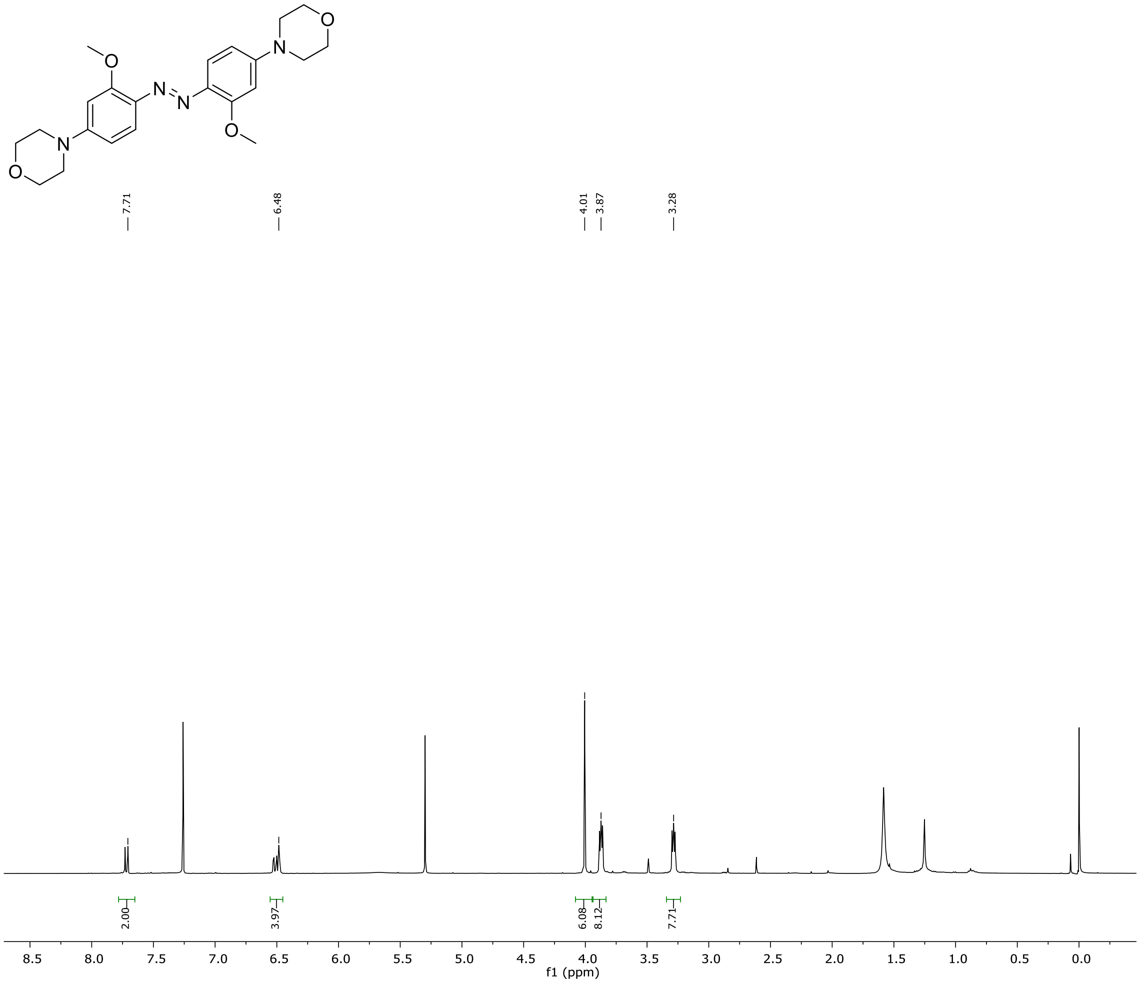

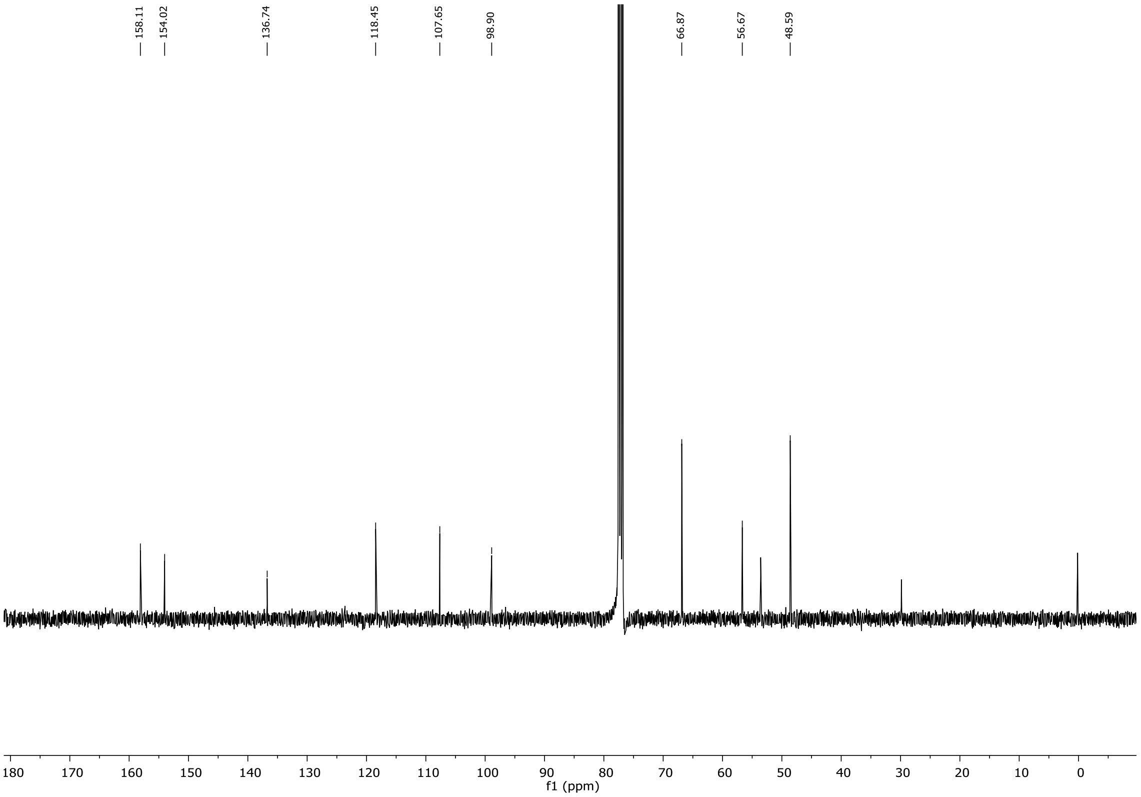
158.11
154.02
136.74
118.45
107.65
98.90
66.87
56.67
48.59
180 170 160 150 140 130 120 110 100 90 80 70 60 50 40 30 20 10 0
f1 (ppm)

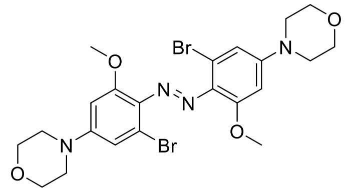
O
N
Br
N
N
Br
O
N
O
O

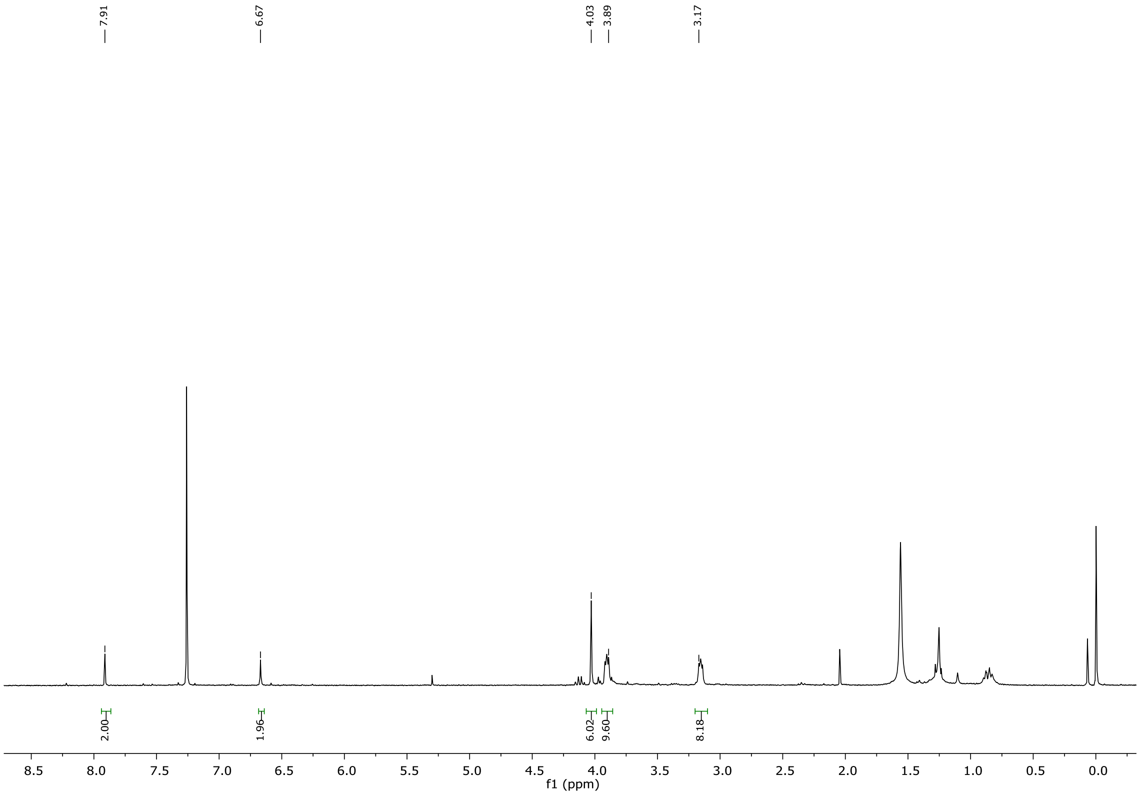
7.91
6.67
4.03
3.89
3.17
2.00
1.96
6.02
9.60
8.18
8.5
8.0
7.5
7.0
6.5
6.0
5.5
5.0
4.5
4.0
3.5
3.0
2.5
2.0
1.5
1.0
0.5
0.0
f1 (ppm)

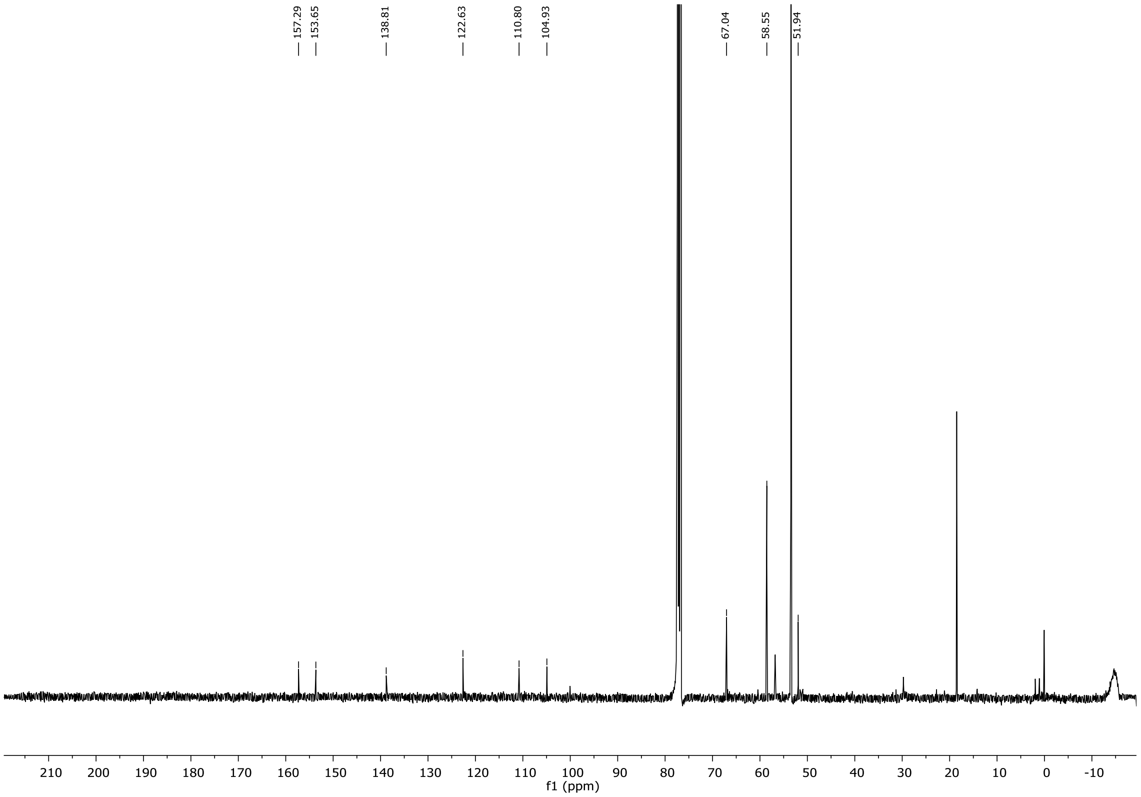
157.29
153.65
138.81
122.63
110.80
104.93
67.04
58.55
51.94
210 200 190 180 170 160 150 140 130 120 110 100 90 80 70 60 50 40 30 20 10 0 -10
f1 (ppm)

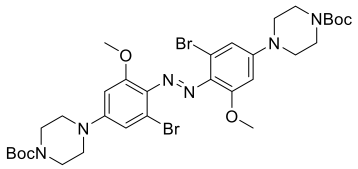

NBoc
Br
O
N
N
N
Br
O
BocN

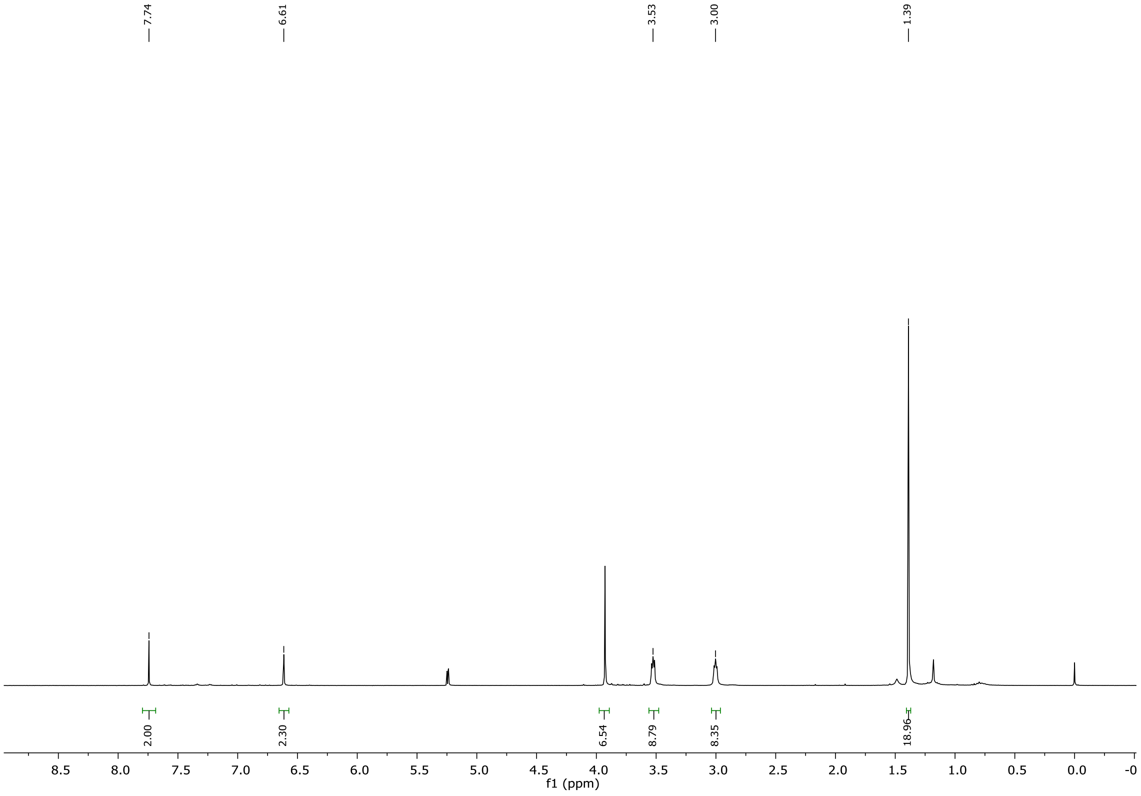

7.74
6.61
3.53
3.00
1.39
2.00
2.30
6.54
8.79
8.35
18.96
8.5
8.0
7.5
7.0
6.5
6.0
5.5
5.0
4.5
4.0
3.5
3.0
2.5
2.0
1.5
1.0
0.5
0.0
-0
f1 (ppm)

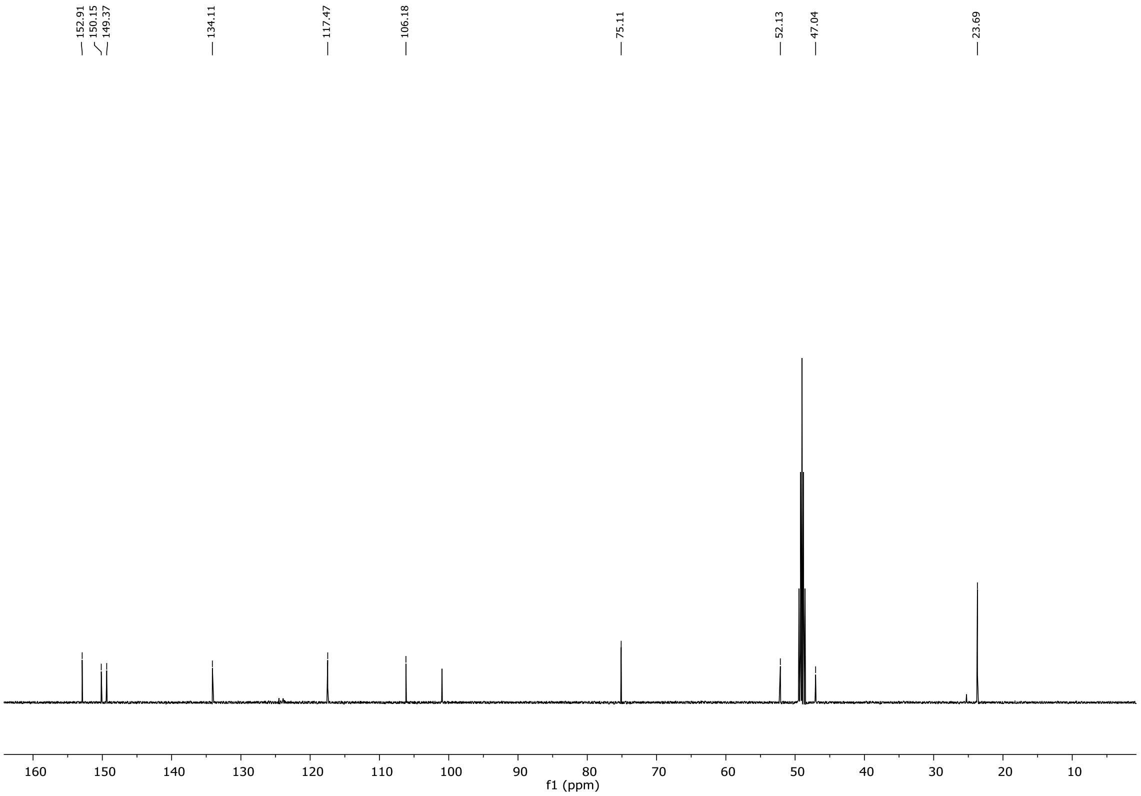
152.91
150.15
149.37
134.11
117.47
106.18
75.11
52.13
47.04
23.69
160 150 140 130 120 110 100 90 80 70 60 50 40 30 20 10
f1 (ppm)

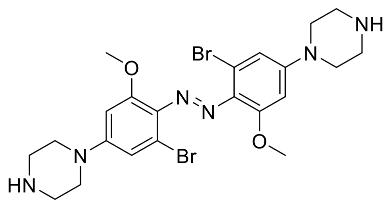
Br
NH
O
N
N
N
HN
Br
O

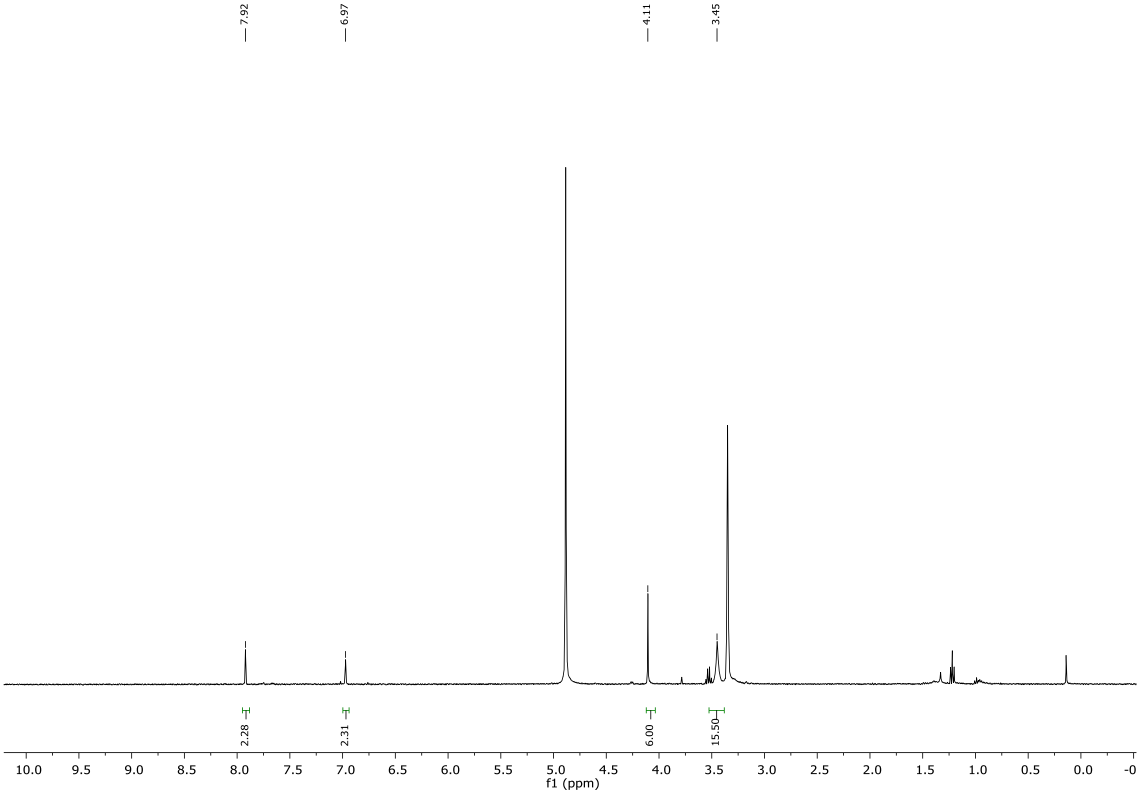
7.92
6.97
4.11
3.45
2.28
2.31
6.00
15.50
10.0 9.5 9.0 8.5 8.0 7.5 7.0 6.5 6.0 5.5 5.0 4.5 4.0 3.5 3.0 2.5 2.0 1.5 1.0 0.5 0.0 -0
f1 (ppm)

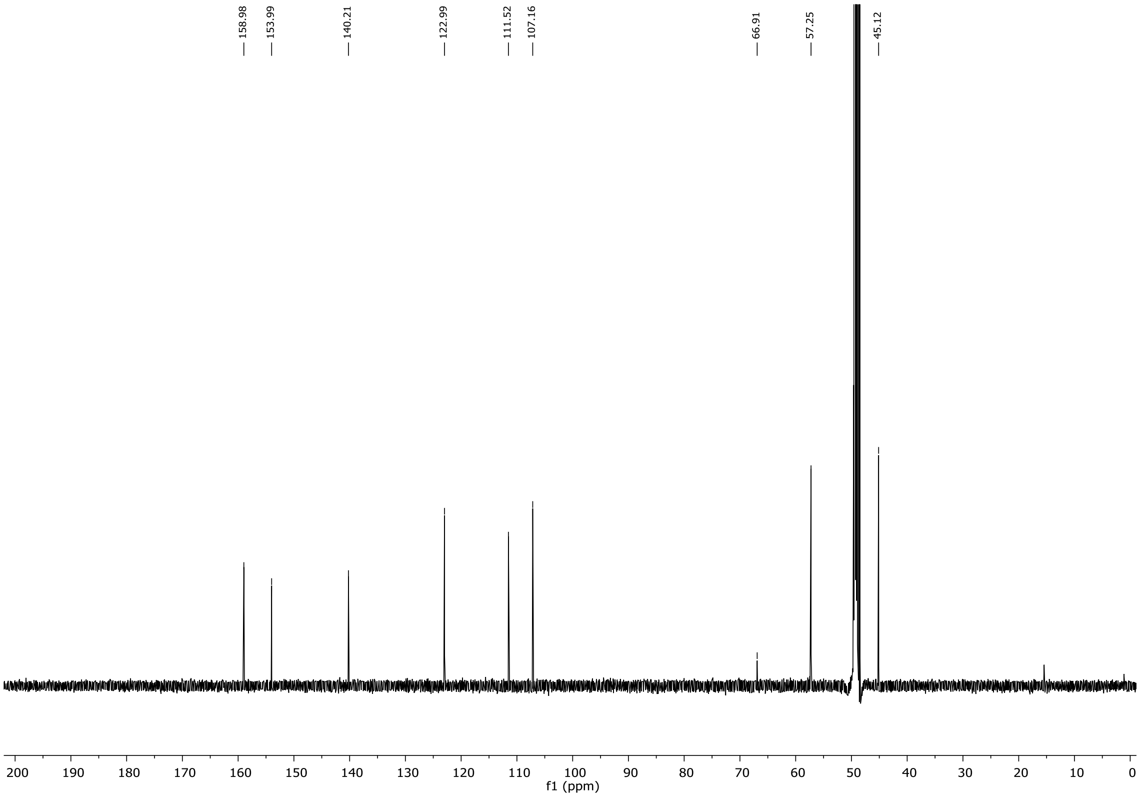
158.98
153.99
140.21
122.99
111.52
107.16
66.91
57.25
45.12
200
190
180
170
160
150
140
130
120
110
100
90
80
70
60
50
40
30
20
10
0
f1 (ppm)

Computational Chemistry

Cartesian coordinates for:

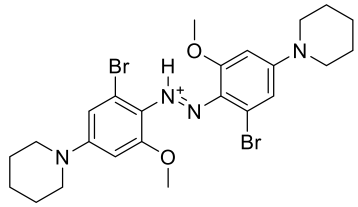


```
63
protonated_6 mem  0.000000
C -2.4550 -1.0500 -0.2060
C -4.0790  1.2300 -0.2700
C -1.8190  0.2440 -0.1950
C -3.8250 -1.1950 -0.2430
C -4.6940 -0.0610 -0.2800
C -2.7150  1.3610 -0.2300
H -4.2370 -2.1900 -0.2220
N  0.4020 -0.3810 -0.1250
C  1.7760 -0.1570 -0.0830
C  4.6350  0.0990  0.0010
C  2.3990  1.1270 -0.0570
C  2.6390 -1.2760 -0.0640
C  4.0110 -1.1780 -0.0220
C  3.7880  1.2340 -0.0190
H  4.2200  2.2200 -0.0470
N -6.0440 -0.2020 -0.3270
C -7.0020  0.9100 -0.1840
H -7.6350  0.9220 -1.0820
H -6.4840  1.8640 -0.1430
C -6.7430 -1.4940 -0.4410
H -6.0380 -2.2970 -0.6400
H -7.3980 -1.4260 -1.3210
N  6.0040  0.2140  0.0760
C  6.6480  1.5210  0.2680
H  5.9750  2.1690  0.8290
H  6.8370  1.9930 -0.7110
C  6.8910 -0.7940 -0.5370
H  6.3690 -1.7450 -0.6230
H  7.1250 -0.4660 -1.5640
C  8.1910 -0.9740  0.2510
H  8.8230 -1.6940 -0.2800
H  7.9610 -1.4040  1.2340
C  7.9620  1.3900  1.0530
H  7.7270  1.0890  2.0820
H  8.4290  2.3800  1.1050
C  8.9120  0.3670  0.4220
H  9.8080  0.2480  1.0400
H  9.2510  0.7300 -0.5590
C -7.5870 -1.7790  0.8100
```

```
H -8.1130 -2.7310  0.6720
H -6.9200 -1.8990  1.6740
C -7.8680  0.7200  1.0720
H -7.2260  0.8010  1.9590
H -8.5930  1.5400  1.1240
C -8.5830 -0.6380  1.0600
H -9.1160 -0.7960  2.0040
H -9.3430 -0.6420  0.2670
N -0.5020  0.5360 -0.1590
H  0.1160 -1.3670 -0.1290
O -1.5990 -2.1050 -0.1660
O  1.5800  2.1940 -0.0920
C  2.1330  3.5130 -0.0640
H  2.7070  3.6790  0.8540
H  2.7610  3.6960 -0.9430
H  1.2740  4.1820 -0.0840
C -2.1060 -3.4490 -0.1770
H -2.7250 -3.6330  0.7060
H -2.6740 -3.6370 -1.0930
H -1.2260 -4.0890 -0.1500
H  4.5920 -2.0860  0.0320
H -4.6710  2.1300 -0.3130
Br  1.8990 -3.0400 -0.0830
Br -1.9990  3.1130 -0.2330
```

Calculated HOMO/LUMO for:

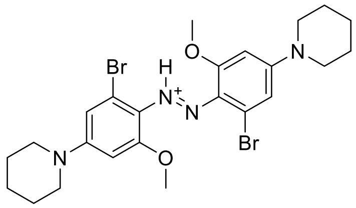

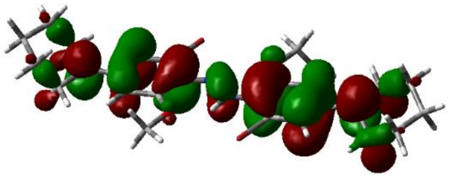

HOMO (sideview)

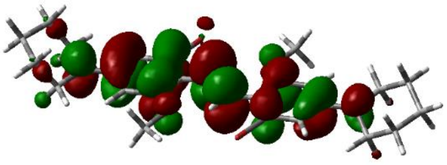

LUMO (sideview)

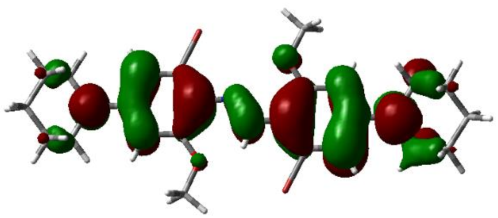

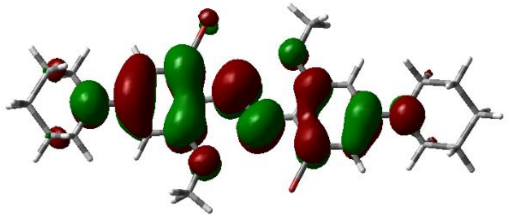

HOMO (topview) LUMO (topview)

Cartesian coordinates for:

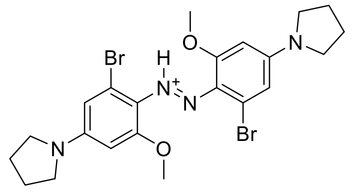


```
57
Molecule Name  0.000000
C -2.4095 -1.1099 -0.0044
C -4.0657  1.1590  0.0093
C -1.7956  0.1964  0.0073
C -3.7794 -1.2735 -0.0133
C -4.6471 -0.1433 -0.0049
C -2.7021  1.3071  0.0131
H -4.2005 -2.2674 -0.0090
N  0.4361 -0.3984  0.0081
C  1.8074 -0.1569  0.0042
C  4.6519  0.1373 -0.0067
C  2.4124  1.1409 -0.0085
C  2.6835 -1.2670  0.0093
C  4.0549 -1.1530  0.0051
C  3.7966  1.2680 -0.0150
H  4.2291  2.2566 -0.0108
N -5.9874 -0.2984 -0.0103
C -6.9489  0.8183  0.0940
H -6.9595  1.3939 -0.8415
H -6.6675  1.4917  0.9089
C -6.6705 -1.6013 -0.1221
H -6.5567 -2.1683  0.8125
H -6.2398 -2.1916 -0.9373
```

N	6.0010	0.2764	-0.0109
C	6.6935	1.5706	-0.1231
H	6.5811	2.1465	0.8069
H	6.2739	2.1629	-0.9435
C	6.9445	-0.8497	0.0976
H	6.6560	-1.5185	0.9151
H	6.9539	-1.4323	-0.8347
C	8.2960	-0.1679	0.3509
H	9.1337	-0.7672	-0.0135
H	8.4387	-0.0097	1.4256
C	8.1571	1.1807	-0.3732
H	8.8531	1.9392	-0.0071
H	8.3310	1.0554	-1.4476
C	-8.1360	-1.2256	-0.3802
H	-8.3054	-1.1017	-1.4554
H	-8.8252	-1.9920	-0.0181
C	-8.2919	0.1202	0.3446
H	-8.4358	-0.0394	1.4188
H	-9.1347	0.7105	-0.0221
N	-0.4804	0.5058	0.0106
H	0.1633	-1.3884	0.0069
O	-1.5369	-2.1518	-0.0019
O	1.5741	2.1935	-0.0091
C	2.1089	3.5200	-0.0215
H	2.7123	3.7090	0.8732
H	2.7048	3.6952	-0.9239
H	1.2405	4.1772	-0.0228
C	-2.0295	-3.5015	-0.0121
H	-2.6270	-3.6974	0.8832
H	-2.6166	-3.6875	-0.9164
H	-1.1424	-4.1324	-0.0105
H	4.6623	-2.0463	-0.0033
H	-4.6924	2.0390	0.0019
Br	-2.0052	3.0652	0.0213
Br	1.9653	-3.0389	0.0178

Calculated HOMO/LUMO for:

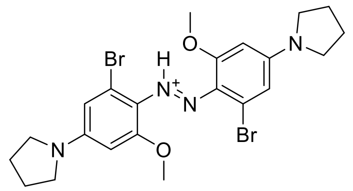

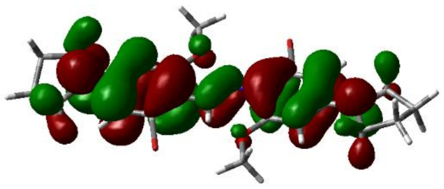

HOMO (sideview)

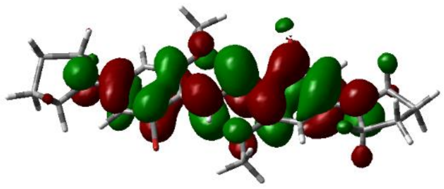

LUMO (sideview)

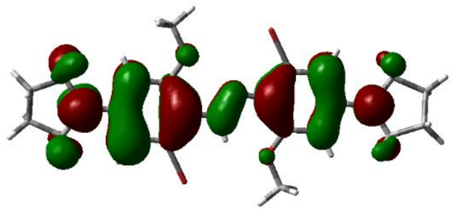

HOMO (topview)

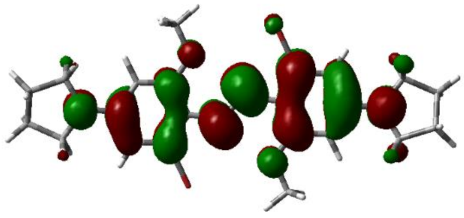

LUMO (topview)
